# Global and Local Processing of Letters and Faces in Children and Adolescents with Typical and Atypical Development

**DOI:** 10.3390/brainsci16010096

**Published:** 2026-01-16

**Authors:** Silvia Primativo, Roberta Daini, Jennifer Pavia, Elisa Fucà, Floriana Costanzo, Cristina Caciolo, Paolo Alfieri, Deny Menghini, Stefano Vicari, Lisa S. Arduino

**Affiliations:** 1 Department of Human Sciences, LUMSA University, 00193 Roma, Italy; 2 Department of Psychology, University of Milano-Bicocca/Milan Centre for Neuroscience (NeuroMI), 20126 Milan, Italy; 3 IRCCS Fondazione Don Carlo Gnocchi ONLUS, 20148 Milan, Italy; 4 Child and Adolescent Neuropsychiatry Unit, Bambino Gesù, Children’s Hospital, IRCCS, 00165 Rome, Italy; 5 Department of Life Science and Public Health, Università Cattolica del Sacro Cuore, 00168 Rome, Italy

**Keywords:** global and local processing, hierarchical letters and faces, Down’s syndrome, Williams’s syndrome, face processing, atypical development

## Abstract

Background/Objectives: this paper investigates the local vs. global visual processing preference in typically developing (TD) children, youth with Down syndrome (DS), and youth with Williams syndrome (WS). In particular, the global precedence effect (GPE) and the global interference effect (GI) have recently been described as two distinct and at least partially independent effects. Methods: in this study, 50 participants (TD = 25, DS = 13, WS = 12) completed two experiments requiring the identification of either the global or local level of hierarchical stimuli, which consisted of letters and schematic faces. For each stimulus type, two separate blocks were conducted, one with the task to focus on the local elements and the other with the task to focus on the global shape. Results: our results indicate that TD children demonstrate a global precedence effect for letters but not for schematic faces, suggesting a developmental modulation of configural processing. In contrast, both DS and WS groups showed a global processing bias for schematic faces and a significant global interference effect in both conditions, likely reflecting deficits in inhibitory control. Conclusions: these findings challenge the notion that DS and WS individuals can be classified strictly as global or local processors, respectively, emphasizing the influence of stimulus type and cognitive demands. Implications for neurodevelopmental research and clinical interventions are discussed.

## 1. Introduction

In perceiving objects of the visual scenes, we build representations of global percepts that are composed of local details (e.g., the trees in a forest), and one important cognitive function of the human brain is to integrate information of both these levels. The question of whether we first perceive parts or the whole has long been debated. Hebb (1949) [1] proposed that perception initially relies on analyzing individual components, which are later integrated into a complete shape. On the other hand, the assumptions formulated by Gestalt theorists suggested that perception is dominated by awareness of the complete whole and that only after its analysis would the whole be perceptually segmented into constituents (the whole is more than its parts). In line with this global processing priority, the more recent Reverse Hierarchy Theory [2] proposes that a first-step automatic process of building a global representation of the external environment (vision at a glance) is needed for a second-step attentional orientation that guides fine-grain (local) visual representations. All these theories converge in supporting the view that, initially, a global analysis of the perceptual scene is achieved and that single elements are analyzed only in a second step.

Recent research has extensively investigated (1) which of the levels (global or local) is perceived first, (2) how the prevalence of one of the two perceptual analyses could be modulated by the type of stimulus, and (3) whether and how the local or global preference may change during the lifetime.

Concerning the first research question, a very useful paradigm for studying multiple levels of interaction between local and global levels is the task introduced by Navon [3,4], who created forms containing hierarchical levels. These forms are composed of small letters whose spatial location and configuration create a big letter. The small letter represents the local level, whereas the big letter represents the global one. The intriguing manipulation by Navon was that small and big letters could be the same (a big E composed of small Es, *congruent condition*) or different (a big E composed of small Ss, *incongruent condition*). The participant’s task was to respond to one level while ignoring the other unattended level. A global precedence effect (GPE) has been demonstrated in such a task under a wide variety of conditions (e.g., [5]). The GPE has been described as characterized by two main components: (i) a global advantage effect (GA), consisting of a faster response time (RT) and a higher accuracy at identifying the global level of the stimulus than the local one; and (ii) a larger global interference effect (GI) in the processing of incongruent stimuli from the global to the local elements than the opposite.

Since these two effects (global advantage and global interference) have often been reported together in the literature [6,7], it has been assumed that they were a valid measure of the order in which local and global levels of structure are processed, supporting the view that global-level processing precedes local-level processing.

However, further evidence questioned this assumption, in that it was shown that these two effects can be affected differently by various factors. Not only the stimulus size and category (e.g., [7]), but also the stimulus duration have been listed among the factors impacting the interference or the global advantage effects, even though some results are contradictory [8,9]. Taken together, these results challenge the hypothesis that the global advantage and the global interference may reflect the order of a coarse-to-fine temporal dynamic processing in visual object recognition, suggesting that the initial processing strategy may depend on several factors, among which is stimulus type.

Indeed, this is strictly connected to the second interesting point raised in the literature, namely whether the global preference effect, reflected by the above-mentioned effects (global advantage and global interference), may be modulated by the type of stimulus. Generally, the two types of stimuli that have been mostly contrasted have been faces and letters since, according to Farah’s theory, letter and face stimuli enhance a by-part or configural analysis, respectively (see [10]). In [9], for example, participants demonstrated a stronger local processing advantage when viewing letter stimuli and a greater global interference when processing schematic faces, suggesting that the latter are more likely to be processed holistically, with attention directed toward the overall configuration rather than individual features.

Regarding the third research question, a lot of the work on the developmental trajectory of perceptual organization in humans has been performed in recent years, sometimes with contradictory results. In a recent study [11], the authors demonstrated that a local precedence effect appears to dominate perceptual organization until adolescence, when a gradual transition begins for visual perception to become organized around more global or configural aspects of the input. This finding is consistent with several other studies reporting the late development of global processing [12,13,14,15,16]. However, such results are in contradiction with the ones reporting an early sensitivity to global information in infants and children [17,18,19]. A model has been proposed where the global-processing advantage follows a U-shaped pattern of development [20]. In other words, while in infancy the global preference is dominant, it changes during development due to the cognitive demands for integrating local features until it becomes again more globally oriented. In this vein, it has been reported [21] that hierarchical forms processing underwent a protracted development characterized by local preference at younger ages (4 to 5 years), with a switch to adult-like global precedence occurring at 9 years of age after a transition period around 6 years of age.

To sum up, the main research questions and theories revolve around different points regarding: (i) whether or not global processing starts first compared to local processing; (ii) whether stimulus type may somehow influence the prevalence of one type of processing above the other; and (iii) whether global prevalence is stable across age or is subject to developmental changes.

In the present study, we aimed to verify the emergence of a global preference with hierarchical stimuli in the developmental age. In particular, we aimed at verifying whether the mechanisms underlying the global preference in the adult population are already developed in primary school children. To this purpose, we used different stimuli, such as letters and schematic faces, for which the literature has reported a differential involvement of a global or a local preferential way of processing. Indeed, according to Farah’s theory, schematic faces and letter stimuli enhance a configural or a by-part analysis, respectively. Moreover, in order to strengthen the hypothesis that the prevalence of one strategy over the other may also depend on stimulus type, we extended our study to two atypical developmental populations that have been documented to use mainly different processing strategies.

Indeed, in order to better understand the global and local processing, some studies have focused on neurodevelopmental disorders, such as Williams syndrome (WS) and Down syndrome (DS), in which local and global processing are purported to be dissociated. In WS, the processing of local details is reported to predominate over the process of global properties [22,23,24,25,26]. In contrast, individuals with DS present with the opposite profile; that is, they often show impairments in processing local details. For example, in an early study, Bihrle and collaborators [27] found a double dissociation between individuals with DS and WS. However, more recent studies failed to report such a strong dissociation between the two groups. In their 2016 study, D’Souza and others [28] conducted an in-depth examination of local and global processing across different modalities and levels of processing in individuals with WS and DS. The study’s findings challenge the simplistic categorization of these neurodevelopmental disorders as being either local or global processors. Instead, the processing preferences of individuals with WS and DS might be influenced by the specific tasks and modalities employed.

In the present study, we used hierarchical configurations (Navon paradigm), which allowed us to focus on the identification of the small (local) or the big (global) stimulus. To better investigate the complexity of local and global processing, we also considered the use of congruent and incongruent small-big associations, which allowed us to reveal an indirect effect of unattended stimuli over the attended ones, as well as a further or different effect of precedence for the local and global levels. By adopting letters and schematic faces as stimuli, we collected data on three groups of participants: children with typical development (TD), youths with DS, and youths with WS. In TD children, we expected to obtain the GPE, amplified with schematic faces compared to letter stimuli, on the basis of the literature on larger holistic processing for schematic faces as compared to letters [10]. Moreover, we expected that their GPE should be reduced compared to the adult population [9] since they are still in a developmental stage and thus their cognitive system is still maturing.

Regarding the participants with atypical development, we expected that youths with DS should present a GPE for letters, given their preference for global processing. By contrast, the effect might be smaller for schematic faces. Indeed, multiple studies showed that DS individuals exhibit atypical development in holistic face processing and in processing the spatial relationships between facial features [29,30]. Furthermore, we expected an enhanced congruency effect for all incongruent conditions because of a general inhibitory deficit (e.g., [31]).

On the contrary, we expected that youth with WS would not show a GPE effect for letters, given their predominant local processing. However, we hypothesize that the effect might arise when processing schematic faces. Research into face processing in individuals with WS has revealed both typical and atypical patterns. For instance, ref. [32], by using the whole–part paradigm, reported that individuals with WS processed faces holistically, similar to controls. On the other hand, Riby et al. (2011) [33] showed that individuals with WS struggle to recognize faces composed of non-facial elements, suggesting differences in holistic face processing.

Finally, we expected a global interference effect in WS and DS due to the non-optimal inhibitory systems already described in the literature in both clinical populations [34,35,36,37,38].

Overall, in the following experiments we will assess: (1) the tendency of TD children to visually process different types of stimuli at the global or local level and (2) the plausibility of considering DS and WS individuals as global vs. local processors, respectively, or whether their processing patterns are more complex and strongly modulated by stimuli features and by a more general cognitive ability to inhibit the irrelevant information.

## 2. Materials and Methods

### 2.1. Participants

Inclusion/exclusion criteria for TD children were age between 6 and 11 years, lack of sensory/neurological/neuropsychological conditions, and the ability to recognize the letters. Inclusion/exclusion criteria for youth with DS and WS were age between 6 and 18 years and a lack of neurodevelopmental comorbidities or other neurological conditions.

Participants with DS and WS were recruited among patients referred for a clinical and neuropsychological evaluation/follow-up evaluation at the Child and Adolescent Neuropsychiatry Unit of the Bambino Gesù Children’s Hospital in Rome between September 2021 and December 2023. Each patient underwent a thorough neuropsychiatric medical evaluation and a physical examination, the results of which are included in the patient’s report.

Twenty-five TD children (12 F) without known neurological conditions participated in the study, thirteen (8 F) youth with DS, and 12 (4 F) participants with WS also took part in the study. Mean chronological age, mental age, average verbal and non-verbal IQ are reported in Table 1. The two clinical populations’ mental ages (IQ value measured by using the Wechsler scales × chronological age/100) were matched to each other and with the TD chronological age (*p* > 0.05).

Ethical approval was obtained from both the Bambino Gesù Children’s Hospital (protocol number: 2157_OPBG_2020) and LUMSA University (CERS_Commettee evaluation_28-01-2020). We obtained a signed informed consent from the parents of all the participants, and verbal assent was obtained from the young participants themselves. All experiments were performed in accordance with relevant guidelines and regulations.

### 2.2. Apparatus and Procedure

At the time of the scheduled 2-consecutive-day neuropsychiatric follow-up visit, youth with DS and WS were subjected to a clinical and neuropsychological evaluation. Their parents were asked to participate. If parents accepted and participants met the criteria, they took part in the experiment. The experimental session lasted 30–45 min, and it was conducted in a quiet and well-lit room. Participants were seated in front of a 15-inch computer monitor with a resolution of 1920 × 1080 pixels and a refresh rate of 60 Hz. The viewing distance was constant at 57 cm. The experiment was controlled by the SR Research Experiment Builder software, 2.2.2 version (SR Research Ltd., Kanata, ON, Canada). The same apparatus was used for all the experiments described below.

For each stimulus type, two separate blocks were conducted, one focusing on the local elements and the other on the global shape.

The order of presentation of the two blocks was counterbalanced across participants. We adopted the same stimuli and procedure used in investigating the adult population [9]. For the sake of clarity, we report the details below.

### 2.3. Experiment 1a—Hierarchical Letter Processing

**Figure 1:**
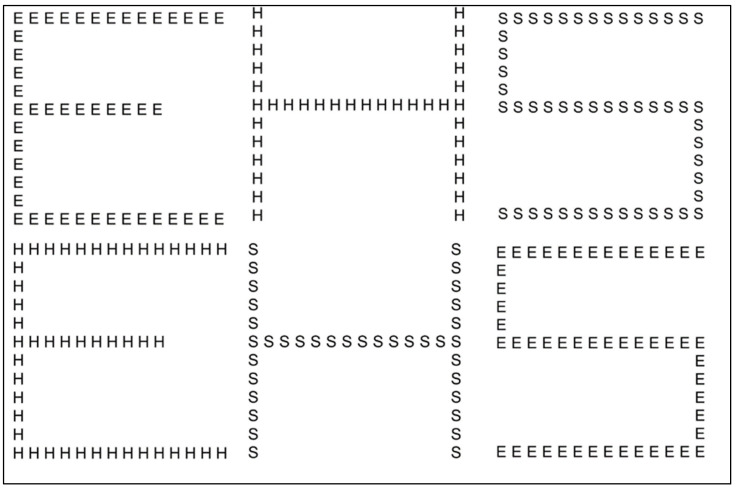
Hierarchical letters. Congruent stimuli are reported in the upper line, incongruent stimuli in the lower line.

In Experiment 1a, hierarchical stimuli were utilized, as depicted in Figure 1, comprising big configurations of three distinct letters (E, H, and S, rendered in Courier New font style), which could be composed of smaller letters, the same (congruent) or different (incongruent). All possible permutations of congruent and incongruent stimuli (i.e., where the identity of the big and small shapes matched or differed, respectively) were presented, yielding a total of nine unique combinations. The overall dimensions of the stimulus were 15 × 15°, with the size of the small letters measuring 1.2 × 1.2° and an inter-letter spacing of 0.6° (measured from border to border).

Two separate blocks were conducted for the two tasks, one focusing on the local level and the other on the global level. In the local block, participants were instructed to identify the smaller, local letters, whereas in the global block they were asked to identify the larger, global letters. The sequence of presentations for the two blocks was counterbalanced across participants. Each block comprised 45 trials: 15 congruent and 30 incongruent. Participants underwent a familiarization phase with printed versions of the stimuli, and the computerized experimental session began only after participants correctly responded to three consecutive stimuli in both the global and local blocks.

Subsequently, a fixation cross, spanning 0.5° of visual angle, appeared at the center of the screen for 300 ms, followed by the hierarchical stimulus for 1000 ms. Participants were asked to identify the global or the local letter, conditions that further on we will call “global task” and “local task”, and were instructed to respond as fast and as accurately as possible by pressing designated keys on a keyboard (patches were placed on the H, J, and K keycaps of the keyboard, representing the letters H, S, and E, respectively). Accuracy and response time were recorded and treated as dependent variables.

### 2.4. Experiment 1b—Hierarchical Schematic Face Processing

**Figure 2:**
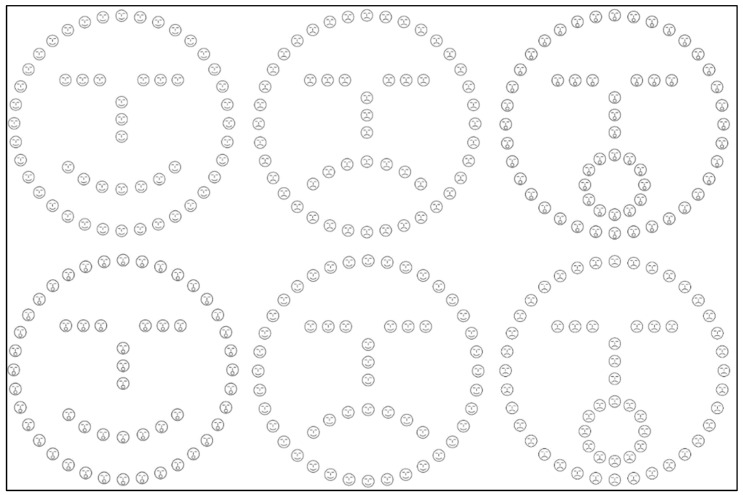
Hierarchical schematic faces. Congruent stimuli are reported in the upper row; incongruent stimuli are reported in the lower row.

Conversely, in Experiment 1b, schematic faces were utilized as hierarchical stimuli (see Figure 2). The big stimuli were stylized face representations displaying emotions such as happiness, sadness, or surprise. Correspondingly, the constituent small faces forming the big one could also exhibit the same (congruent) or different emotion (incongruent). Mirroring Experiment 1, all feasible combinations of congruent and incongruent stimuli were employed. The dimensions of the stimuli remained consistent with those used for letters, with the big face occupying an area of 15 × 15° and the small faces measuring 1.2 × 1.2° each. Furthermore, the distance among small elements was maintained at 0.6°. Stimulus generation was achieved using Adobe Photoshop software (CC 2015, version 16).

Similarly to the experiments with letters, participants underwent a preliminary familiarization phase with printed versions of the stimuli, and the experimental session started only after participants achieved three consecutive correct responses on both the global and local stimuli.

Following the familiarization phase, the experiment began with the presentation of a fixation cross measuring 0.5° of visual angle at the center of the screen for 300 ms, followed by the appearance of the stimulus for 1000 ms. Participants were instructed to identify the emotional expression of either the global or local schematic face and to respond as fast and accurately as possible by pressing designated keys on a keyboard (patches indicating happiness, sadness, and surprise were attached over the H, J, and K keys, respectively). Accuracy and response time were recorded as performance metrics. As for experiment 1a, each condition (global task or local task) comprised 45 trials: 15 congruent and 30 incongruent.

## 3. Results

### 3.1. Statistical Analysis

The JAMOVI software (version 2.3.28) was used for all the analyses. Separate repeated measures ANOVAs were run on accuracy and response times for letters and schematic faces with Congruency (congruent vs. incongruent) and Task (global vs. local) as within-subject factors and Group (WS vs. DS vs. TD) as a between-subject factor. *p*-values equal to or below 0.05 were considered statistically significant and were further tested by direct *t*-test comparisons. Effect sizes have been evaluated and estimated in terms of partial eta-squared values (η^2^p). The statistically main effects of Group and the interactions were further explored through Tukey post hoc comparisons.

### 3.2. Letters—Accuracy

Main effects of Task (F(1,47) = 6.8, *p* = 0.01, η^2^p = 0.13), Congruency (F(1,47) = 74.4, *p* < 0.001, η^2^p = 0.6), and Group (F(2,47) = 30.3, *p* < 0.001, η^2^p = 0.56) emerged as statistically significant. Results indicated that participants were more accurate with global vs. local stimuli (89.6% vs. 85.6%) and with congruent vs. incongruent stimuli (92.5% vs. 82.7%). Overall, TD children were more accurate (96.9%) than participants with DS (78.4%, *p* < 0.001) and WS (78.8%, *p* < 0.001), while the two clinical groups did not differ from each other (*p* = 0.99). The main effects were further clarified by the significant interactions Congruency × Group (F(2,47) = 17.87, *p* < 0.001, η^2^p = 0.43) and Task × Congruency (F(1,47) = 4.9, *p* = 0.03, η^2^p = 0.095). The Congruency × Group interaction revealed that for TD children there was no difference between the congruent vs. incongruent condition, both nearly at ceiling (98.7% vs. 94.8%, *p* = 0.3), while both participants with DS and WS were significantly more accurate with the congruent vs. the incongruent conditions (DS: 89.5% vs. 67.2%, *p* < 0.001; WS: 83.1% vs. 74.1%, *p* = 0.01). Also, the interaction revealed that, in the congruent condition, TD children were significantly more accurate than both participants with DS and WS (both *p* < 0.01), while the two clinical groups did not differ from each other (*p* = 0.3).

**Figure 3:**
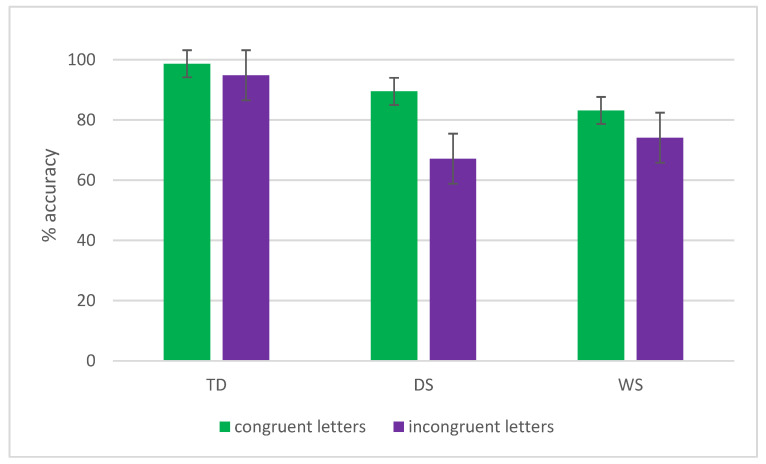
The Congruency × Group interaction in terms of letter accuracy is reported. Bars represent standard errors. Groups: TD (typical developmental children), DS (participants with Down syndrome), and WS (participants with Williams syndrome).

Similarly, in the incongruent condition, TD children were significantly more accurate as compared to both participants with DS and WS (both *p* <0.001), while DS and WS did not differ from each other (*p* = 0.6). The results of this interaction are also reported in Figure 3. The Task × Congruency interaction showed that when letters are congruent, individuals are more accurate with global vs. local (96% vs. 89.1%, *p* < 0.01), while in the case of incongruent letters, global and local stimuli are not different in terms of accuracy (83% vs. 82.3%, *p* = 0.99). All the other interactions were not statistically significant.

### 3.3. Letters—Response Times

The analysis of RT indicated significant main effects of Task (F(1,47) = 18.5, *p* < 0.001, η^2^p = 0.28) and Congruency (F(1,47) = 6.6, *p* = 0.01, η^2^p = 0.12), and a significant interaction Task × Group (F(2,47) = 18.3, *p* < 0.001, η^2^p = 0.4). The main effect of the Group did not reach statistical significance (*p* = 0.2).

**Figure 4:**
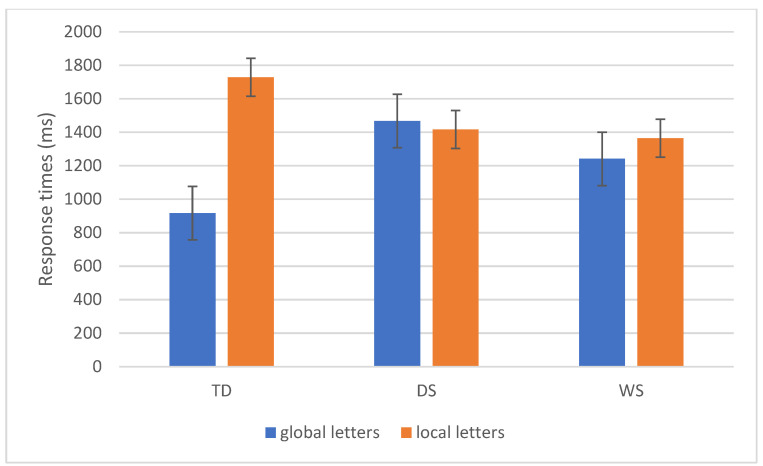
The task × Group interaction in terms of letter reaction times (ms) is reported. Bars represent standard errors. Groups: TD (typical developmental children), DS (participants with Down syndrome), and WS (participants with Williams syndrome).

Participants were faster with global vs. local letters (1138 ms vs. 1560 ms) and with congruent vs. incongruent stimuli (1314 ms vs. 1383 ms). The Task × Group interaction revealed that TD children have a GPE, with faster RTs for global vs. local stimuli (917 ms vs. 1728 ms, *p* < 0.001), while participants with DS and WS have similar RTs for global and local letters (DS: 1467 ms vs. 1417 ms, *p* = 0.99; WS: 1241 ms vs. 1365 ms, *p* = 0.94). The interaction also revealed that TD children are faster than participants with DS and WS in processing global letters (*p* < 0.001 and *p* = 0.03, respectively), but there are no differences between the three groups in terms of processing times for local letters (all *p* > 0.05). Results are reported in Figure 4.

### 3.4. Schematic Faces—Accuracy

Statistics revealed main effects of Task (F(1,47) = 19.4, *p* < 0.001, η^2^p = 0.29), Congruency (F(1,47) = 67.2, *p* < 0.001, η^2^p = 0.59), and Group (F(2,47) = 30.1, *p* < 0.001, η^2^p = 0.56). Overall, global stimuli were more accurate than local stimuli (89.2% vs. 83%), congruent stimuli were more accurate than incongruent ones (91.5% vs. 80.7%), and TD children were more accurate (93.8%) than participants with DS (77.5%, *p* < 0.001) and WS (79.3%, *p* < 0.001).

The statistically significant interactions Task × Group (F(2,47) = 6.03, *p* = 0.005, η^2^p = 0.2) and Congruency × Group (F(2,47) = 6.2, *p* = 0.004, η^2^p = 0.21) further clarified the picture. In particular, we observed that TD children were more accurate in local processing (93.7%) as compared to participants with DS (71.2%, *p* < 0.001) and WS (73.3, *p* < 0.001). Conversely, the three groups processed equally well the global elements (TD: 93.8%, DS: 83.8%, WS: 85.3%, all *p* > 0.05). Moreover, TD children processed equally well global and local schematic faces (*p* = 1), while participants with DS and WS showed an advantage for global vs. local processing (*p* = 0.008 and *p* = 0.02, respectively).

**Figure 5:**
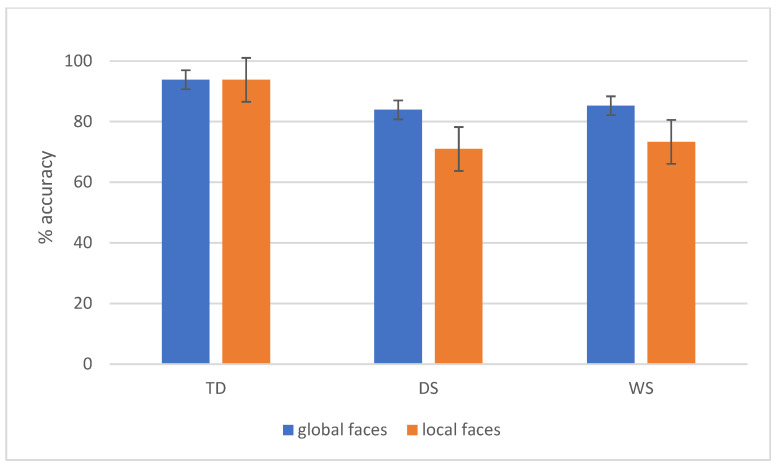
The Task × Group interaction in terms of schematic faces accuracy is reported. Bars represent standard errors. Groups: TD (typical developmental children), DS (participants with Down syndrome), and WS (participants with Williams syndrome).

Results of this interaction are also reported in Figure 5.

**Figure 6:**
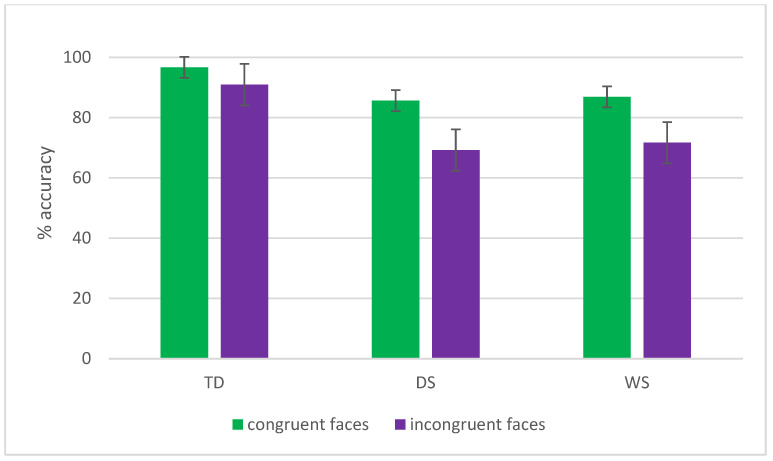
The Congruency × Group interaction in terms of schematic faces accuracy is reported. Bars represent standard errors. Groups: TD (typical developmental children), DS (participants with Down syndrome), and WS (participants with Williams syndrome).

The Congruency × Group effect (reported also in Figure 6) revealed that while for TD participants the difference between congruent and incongruent stimuli was not statistically significant (96.7% vs. 90.9%; *p* = 0.07), participants with DS and WS were significantly more accurate with congruent vs. incongruent stimuli (DS: 85.8% vs. 69.3%, *p* < 0.001; WS: 86.8% vs. 71.7%, *p* < 0.001). TD children were significantly more accurate than participants with DS and WS on both the congruent (*p* = 0.002 and *p* = 0.01, respectively) and incongruent conditions (both *p* < 0.001), while the two clinical groups did not differ from each other in either the congruent or the incongruent conditions (both *p* > 0.1).

### 3.5. Schematic Faces—Response Times

Statistics revealed main effects of Task (F(1,47) = 18.2, *p* < 0.001, η^2^p = 0.28) and Group (F(2,47) = 21.2, *p* < 0.001, η^2^p = 0.48). Global task response time was overall faster as compared to the local task (1500 vs. 1747 ms). Moreover, the group effect indicated that overall TD children (1396 ms) were faster than participants with DS (2073 ms, *p* < 0.01) but not compared to youth with WS (1609 ms, *p* = 0.12). Furthermore, participants with WS were faster than youth with DS (*p* = 0.001).

**Figure 7:**
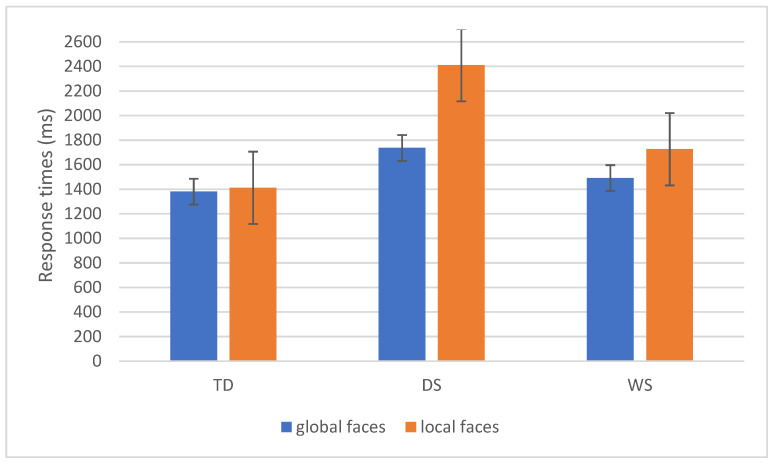
The Task × Group interaction in terms of schematic faces response times (ms) is reported. Bars represent standard errors. Groups: TD (typical developmental children), DS (participants with Down syndrome), and WS (participants with Williams syndrome).

Also, the interaction Task × Group was statistically significant (F(2,47) = 7.3, *p* = 0.002, η^2^p = 0.24). The interaction revealed that while TD children did not differ from youth with WS in either global or local processing (both *p* > 0.1), participants with DS were slower in processing faces globally and locally as compared to TD children (*p* = 0.1 and *p* < 0.001, respectively). Participants with DS were also slower than youths with WS in processing local faces (*p* = 0.009). Results also showed that TD children and youth with WS processed faster global and local schematic faces similarly (*p* = 1 and *p* = 0.6, respectively), while DS were significantly slower in processing local schematic faces (*p* < 0.001). Results are reported in Figure 7.

## 4. Discussion

In the present study, we examined the preference for global or local perceptual processing in typically developing children and atypical youths using the Navon paradigm [3]. Additionally, we investigated whether this preference depends on stimulus type (letters or schematic faces). To explore these aspects, we applied the same paradigm to three groups: TD children and youths with DS and WS.

When letters were used, TD children showed a global advantage, as indicated by the GPE, in terms of both accuracy and response times. On the other hand, both youths with DS and WS showed an advantage for global letter processing in terms of accuracy. Response times were similar for global and local processing, suggesting a lack of a specific letter processing strategy. Nonetheless, the two clinical groups, but not the TD group, exhibited an interference effect, with congruent stimuli being processed more accurately than incongruent ones. One possible account for the pronounced global interference effects observed in the DS and WS groups relates to domain-general inhibitory control mechanisms (e.g., [36,39,40]). In particular, DS and WS have already been shown to have lower performance in response inhibition (go/no-go task) than TD [36,40]; in the latter case, ref. [36] have suggested that it could depend on the dysfunction of the frontostriatal circuits they found in WS. An altered activation of the cingulate cortex, within a more extended alteration pattern, was also in DS infants [41]. However, since inhibitory functions were not directly measured in the present study, this interpretation should be considered speculative and grounded in prior evidence rather than empirical confirmation. Future studies directly assessing executive functions will be necessary to test this account.

Our results also seem to suggest that schematic faces stimuli did not elicit a configural (global) processing strategy in TD children, contrary to what was observed with adults [9] and with what was expected on the basis of Farah’s theory [10].

One speculative interpretation of the absence of a global processing advantage for schematic faces in TD children relates to the perceptual ambiguity of near-face stimuli. Schematic faces may elicit a form of perceptual interference, rather than holistic facilitation, particularly in children. In this respect, previous work has shown that typically developing children can exhibit sensitivity to near-human representations, sometimes described in terms of an “uncanny valley” effect [42]. However, since affective responses were not measured in the present study, this interpretation should be regarded as a tentative hypothesis rather than a definitive explanation. Furthermore, it is important to note that schematic faces do not recruit configural processing in the same manner as real faces [43]. While real-face configural processing involves sensitivity to second-order spatial relations among facial features, schematic faces likely elicit a more general form of global or holistic spatial integration. Thus, in the present study, the term “configural processing” is used to indicate a relative preference for global organization over local feature-based analysis, rather than face-specific configural mechanisms.

The results from the present study also indicate a GPE effect in both our clinical populations, and this might be related to their general cognitive profile. In particular, enhanced social motivation in populations with DS may lead to increased acceptance of near-human representations, leading them to use a preferential global processing strategy to process schematic faces. Similarly, individuals with WS are characterized by hyper sociability and heightened emotional sensitivity to faces. As such, they may not undergo the UV effect that we observed in TD children and thus process schematic faces holistically, as is the case for adults [9].

Finally, and as for letters, both DS and WS youths also showed the global interference effect when accuracy was considered. The emergence of a great global interference effect in these clinical populations was expected on the basis of their general difficulties in inhibiting the incongruent stimulus at the level that should be unattended [31].

We did not confirm the results, indicating that individuals with WS present a local advantage effect (e.g., [22,23]). We found a partial stimulus-type specificity since the GPE is stronger for schematic faces (concerning both accuracy and response times) as compared to letters, where the effect only concerns accuracy. It is interesting to note that participants with DS and WS reported a global interference effect for both letters and schematic faces, which was not evident for TD children. Grounded on prior evidence, this effect might be linked to an inhibition deficit in both populations, which might obstruct adherence to the local/global task when the big and small elements of the hierarchical figures are incongruent. However, the present data do not allow us to disentangle perceptual from executive contributions.

Our results add to the literature on global-local processing in the life cycle, suggesting that the development of the mechanisms underlying the GPE is flexible and changeable on the basis of the characteristics of the processors, the task, and the type of stimuli to be processed. Indeed, this further challenges the hypothesis that global advantage and global interference reflect a coarse-to-fine temporal dynamic in visual object recognition. Instead, they suggest that the initial processing strategy may depend on multiple factors, including the type of stimulus.

Our study is also in agreement with the literature on neurodevelopmental conditions, prompting more nuanced definitions of individuals with DS and WS as global vs. local processors.

From an applicative and clinical perspective, our results suggest that interventions and support strategies should be tailored to the individual’s specific processing tendencies, considering the variability across different contexts and tasks.

Several limitations of the present study should be acknowledged.

First, the sample size, particularly for the DS and WS groups, was relatively small. Although this is common in research involving rare neurodevelopmental conditions, the limited number of participants reduces statistical power and may limit the generalizability of the findings, increasing the risk of type 1 error. Future studies would benefit from larger and more diverse samples to confirm and extend the present results.

Second, although the mental ages of the clinical groups were matched to the chronological age of the TD group, the wide chronological age range in the DS and WS samples may have introduced additional variability related to attentional or experiential factors. This limitation is inherent to research on rare neurodevelopmental conditions and reflects a trade-off between cognitive matching and chronological homogeneity.

Third, the study relied on only one type of hierarchical stimulus per category (letters and schematic faces). While these allowed us to probe different levels of configural processing, the use of schematic rather than naturalistic faces—and only three emotional expressions—may have influenced results, particularly given the sensitivity of children to uncanny or near-human stimuli. Including a broader range of stimuli varying in complexity, emotional valence, and perceptual realism could help determine the robustness of the observed effects.

Finally, while inhibitory difficulties were invoked to explain the global interference effects observed in the DS and WS groups, executive functions were not directly measured. Without independent assessment of inhibition, working memory, or attentional control, such interpretations remain speculative. Future studies should include standardized EF assessments to better account for the contribution of domain-general cognitive processes.

Despite these limitations, the study provides new evidence on the flexibility and variability of global-local processing in typical and atypical development and highlights the need for more nuanced models that integrate stimulus characteristics, cognitive abilities, and developmental factors.

## 5. Conclusions

The use of two types of stimuli allowed us to unveil specific behaviors in the three groups, showing: (1) a global advantage effect for letters but not for schematic faces in TD children; (2) a global advantage effect for faces but not for letters in youths with DS and WS; and (3) a global interference effect with both letters and schematic faces in youths with DS and WS individuals, more consistent for participants with DS.

In accordance with [28], we found that youths with DS and WS cannot be really distinguished along the two dimensions, global and local processing, in that it appears evident also in our study that these biases may depend on various factors, such as the stimulus type and the task.

## Figures and Tables

**Table 1:** Main demographic features for the three groups of participants. Sd = standard deviation; IQ = intellectual quotient. Groups: TD (typical developmental children), DS (participants with Down syndrome), and WS (participants with Williams syndrome).

	TD (N = 25)	DS (N = 13)	WS (N = 12)
Mean age (sd)	9.1 (1.7)	14.4 (2.1)	13.1 (2.2)
Mental age		8.3	7.8
Average IQ (sd)		57.7 (5.4)	59.4 (5.1)
Verbal IQ (sd)		49.3 (11.3)	76.9 (5.2)
Non-verbal IQ (sd)		63.6 (13.6)	54.5 (7.2)

## Data Availability

Data are available on this link: https://osf.io/erbxz/overview?view_only=a6944f521b9b492998afc737b8a73626.
